# Characterization of the chloroplast genome of *Symbiochlorum hainanensis* (Ulvophyceae, Chlorophyta) and its phylogenetic analysis

**DOI:** 10.1080/23802359.2023.2183722

**Published:** 2023-03-25

**Authors:** Fangfang Yang, Yi Huang, Lijuan Long

**Affiliations:** a Key Laboratory of Tropical Marine Bio-resources and Ecology, South China Sea Institute of Oceanology, Chinese Academy of Sciences, Guangzhou, China; b University of Chinese Academy of Sciences, Beijing, P. R. China

**Keywords:** *Symbiochlorum hainanensis*, chloroplast genome, phylogenetic analysis

## Abstract

*Symbiochlorum hainandiae* S.Q. Gong & Z.Y. Li, 2018 is a unicellular green alga belonging to Ulvophyceae, Chlorophyta, and plays important roles in coral reef ecosystem. In this study, high-throughput sequencing technology is used to sequence and assemble the chloroplast genome of *S. hainandiae*. The complete chloroplast genome of *S. hainandiae* was 158, 96 bp with the GC content of 32.86%. A total of 126 genes were identified, including 98 protein-coding genes, 26 tRNA, and 2 rRNA genes. The inverted repeat region was lost in the complete chloroplast genome of *S. hainandiae*. The phylogenetic analysis supports that *S. hainandiae* is a new sister lineage to the genus *Ignatius* within the class Ulvophyceae.

## 1. Introduction

*Symbiochlorum hainandiae* S.Q. Gong & Z.Y. Li, 2018, a unicellular green alga, belongs to Ulvophyceae, Chlorophyta, which is closely related to the genus *Ignatius*. The genus *Symbiochlorium* has been reported firstly by Gong et al. (2018). So far, *S. hainandiae* has been only reported species within genus *Symbiochlorum. Symbiochlorum hainandiae* is considered as a new unidentified green algal, and isolated from bleaching coral species. Gong et al. (2022) has shown that *S. hainandiae* is a thermotolerant alga, which can grow rapidly even at 35 °C. We also have isolated *S. hainandiae* in *Pocillopora damicornis*, and observed similar phenomenon that the alga can grow at high temperature. Therefore, we speculate that *S. hainandiae* may play an important role in coral reefs. However, the chloroplast genome structure of *S. hainandiae* and its phylogenetic position are still unclear. In this study, the first complete chloroplast genome of *S. hainandiae* is investigated.

## 2. Materials

*Symbiochlorum hainandiae* was collected from coral *P. damicornis* in the coast of Sanya City, China (18°21′ N and 109°47′ E)*. S. hainandiae* was isolated using a single cell separation technology, and then cultured in f/2 medium with a light intensity of 80 μmol photons m^−2^ s^−1^. *Symbiochlorum hainandiae* was deposited at the laboratory of South China Sea Institute of Oceanology, Chinese Academy of Sciences, Guangzhou City, Guangdong Province (http://www.scsio.ac.cn/, Fangfang Yang, ycuyang@scsio.ac.cn) under voucher number SY20210606.

## 3. Methods

The genomic DNA was extracted with the modified CTAB protocol (Doyle 1987). After DNA extraction, a library with the insertion size of 350 bp was constructed. Whole genome sequencing was carried out using a Novaseq 6000 sequencer (Illumina, San Diego, CA, USA). In total, the raw data totaled 4.75 G, and the clean data totaled 4.69 G after quality control processing. The coverage was showed in the Figure S1. The guanine-cytosine (GC) content of the clean data was 62.08%, the Q20 value was 97.17%, and the Q30 value was 92.50%, indicating a very high level of data quality for the complete chloroplast genome sequencing and assembly results. Cleaned reads were assembled using the de novo assembler SPAdes v.3.14.1 software. Finally, the assembled chloroplast genome was annotated using the PGA program (Qu et al. 2019). To understand the phylogenetic position of *S. hainandiae*, the complete chloroplast genomes of 19 marine microalga were selected to construct a phylogenetic tree (Turmel and Lemieux 2018). Multiple sequence alignment was performed by MAFFT version 7 software with the FFT-NS-2 strategy (Katoh and Standley 2016). The alignment results were calculated using the maximum likelihood algorithm and the phylogenetic tree was constructed by IQ-TREE 2.0 with 1000 bootstraps (Nguyen et al. 2015).

**Figure 1.**
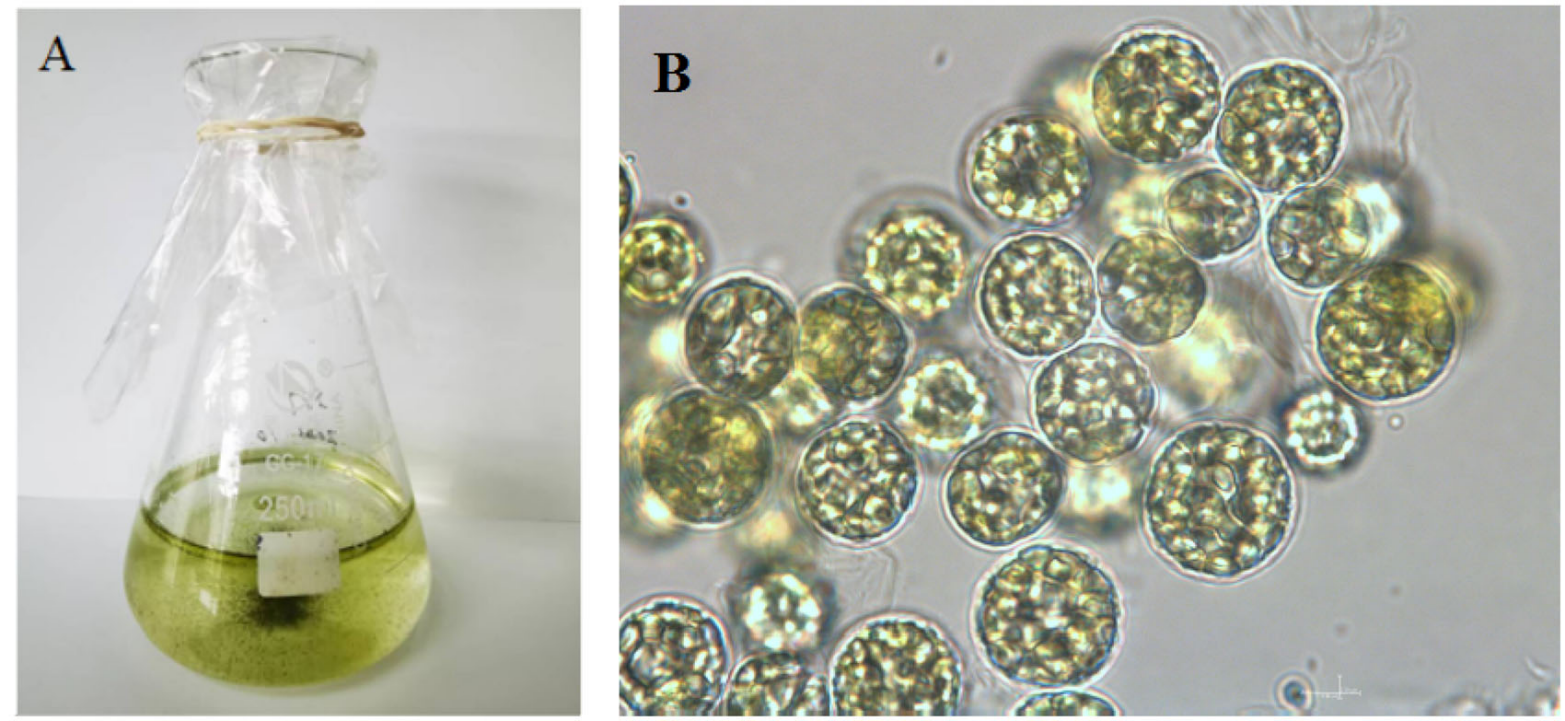
The morphology of *S. hainandiae* (A) in flasks and (B) under a microscope. Photograph was taken by Fangfang Yang.

## 4. Results

**Figure 2.**
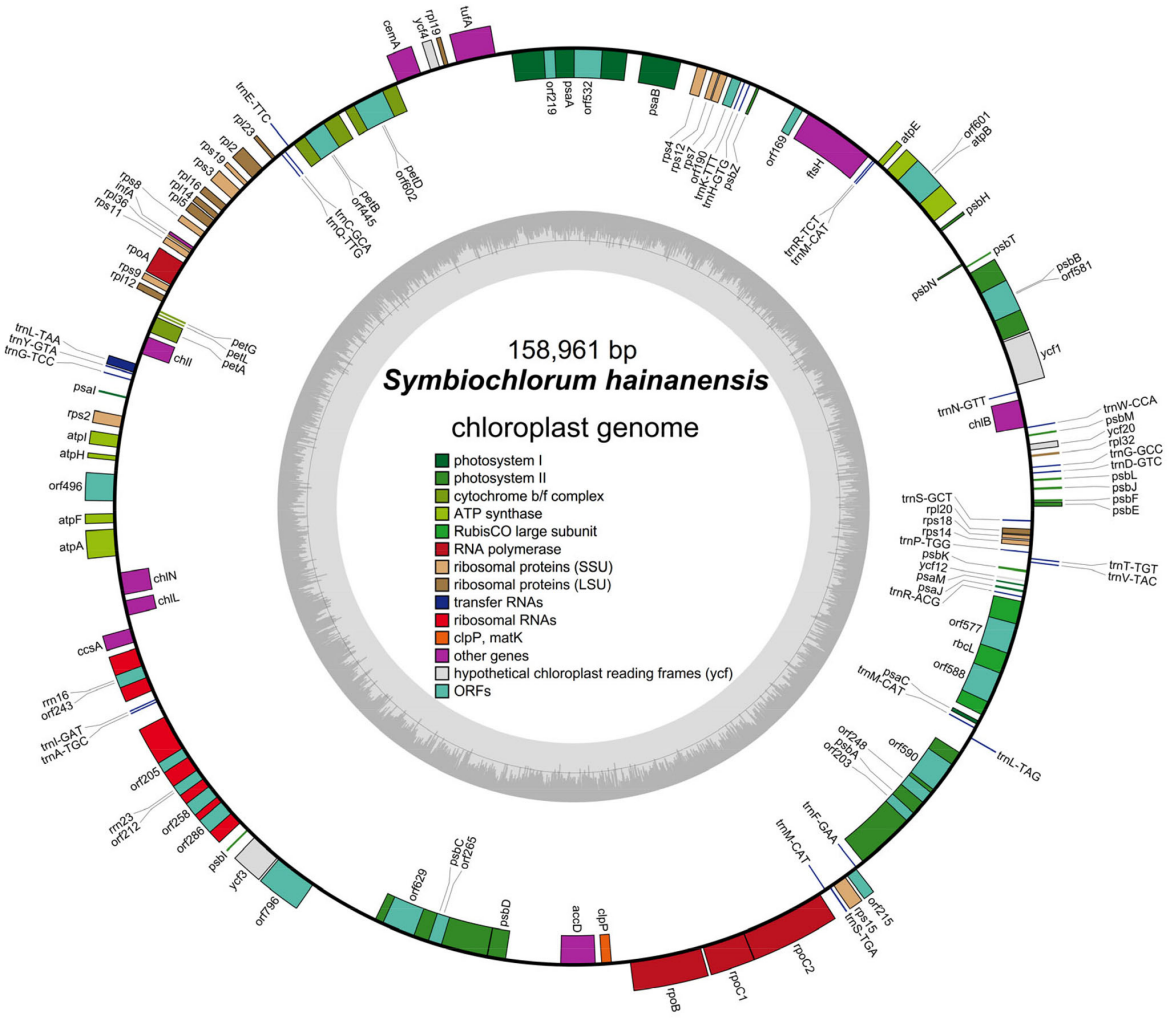
Chloroplast genome map of *S. hainandiae*. Genes are color coded by their function in the legend. Genes on the inner and outer portions of the circle are counterclockwise directions and transcribed in clockwise, respectively.

**Figure 3.**
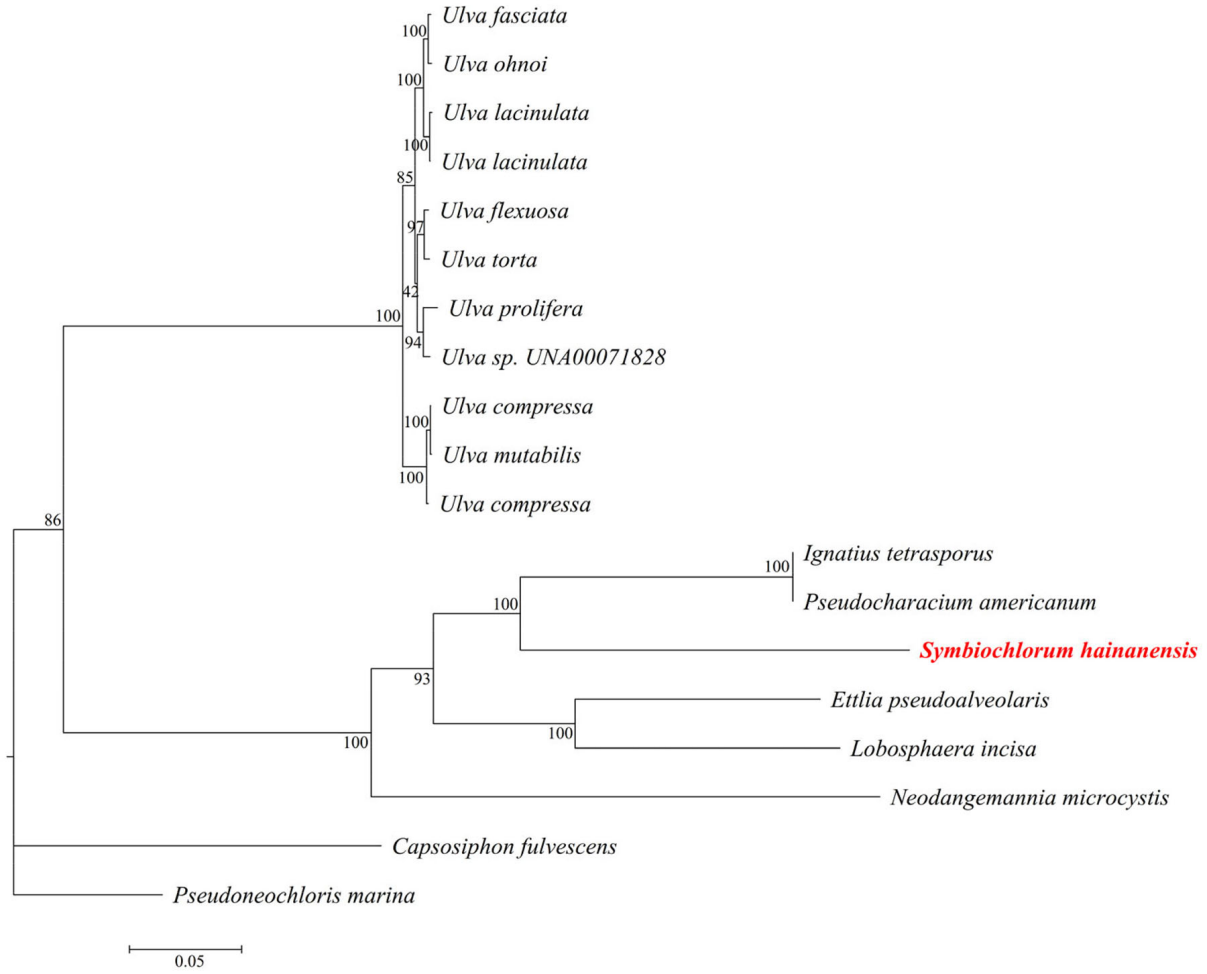
Maximum-likelihood phylogenetic tree based on the chloroplast genome sequences of 19 marine microalgas. The following sequences were used: *Symbiochlorum hainanensis* (ON645925), *Pseudoneochloris marina UTEX 1445* (KY407657.1), *Ulva* sp. UNA00071828 (KP720616), *Ulva fasciata* (NC_029040.1), Ulva flexuosa (KX579943.1), *Ulva torta* (MZ703011.1), *Ulva ohnoi* (AP018696.1), *Ulva compressa* (MW548841.1), *Ulva mutabilis* (MK069584.1), *Pseudoneochloris marina* (KY407657.1), *Lobosphaera incisa* (KM821265.1), *Ulva lacinulata* (MW543061.1), *Ulva lacinulata* (NC_053614.1), *Ulva prolifera* (MN853879.1), *Ettlia pseudoalveolaris* (KM462869.1), *Ignatius tetrasporus* (KY407659.1), *Pseudocharacium americanum* (KY407658.1), *Capsosiphon fulvescens* (NC_039920.1), and *Neodangemannia microcystis* (KY407660.1).

*Symbiochlorum hainandiae* is a type of unicellular phytoplankton, which is green, with a visible chloroplasts, starch granules, pyrenoids, and cell wall (Figure 1). The complete chloroplast genome sequence of *S. hainandiae* was 158,961 bp in length (Figure 2). This chloroplast genome did not have a quadripartite structure due to lacking large inverted repeat regions. This finds consistent with previous results (Turmel et al. 2017). The overall GC content was 32.86%. The chloroplast genome contained 126 genes, including 98 protein-coding genes, 26 tRNA genes, and 2 rRNA genes. The complete chloroplast genome sequence was submitted to GenBank (accession number ON645925). Phylogenetic analysis showed that *S. hainandiae* was a new sister lineage to the genus *Ignatius* within the class Ulvophyceae (Figure 3).

## 5. Discussion and conclusion

In this study, we analyzed the chloroplast genomes information and phylogenetic relationship of *S. hainandiae.* We found that the inverted repeat region was lost in the complete chloroplast genome of *S. hainandiae*. This study provided valuable information for studying phylogenetic relationships within the class Ulvophyceae. Moreover, our study will focus on finding more algal species belonging to *Symbiochlorum*.

## Supplementary Material

Supplemental Material

## Data Availability

The genome sequence data that support the findings of this study are openly available in GenBank of NCBI at (https://www.ncbi.nlm.nih.gov/) under the accession no. ON645925. The associated BioProject, SRA, and Bio-Sample numbers are PRJNA844570, SRR19521218, and SAMN28834117, respectively.
