# Supplementary figures and images for: Characterization of the chloroplast genome of Symbiochlorum hainanensis (Ulvophyceae, Chlorophyta) and its phylogenetic analysis
Source: Mitochondrial DNA B Resour. 2023 Mar 25;8(3):422–5. doi: 10.1080/23802359.2023.2183722 (PMC10044315; doi:10.1080/23802359.2023.2183722)

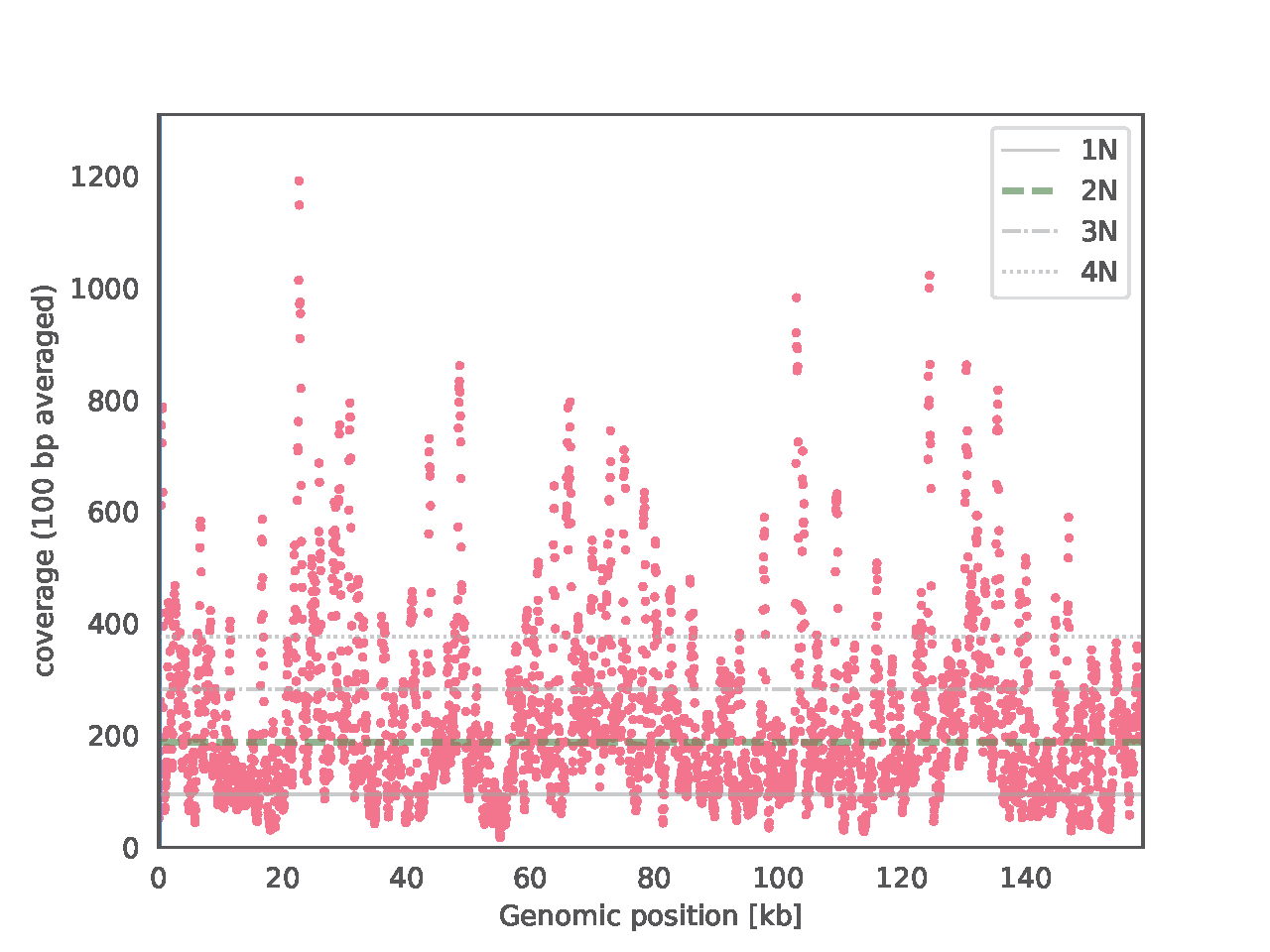

Supplement: Supplemental Material [file TMDN_A_2183722_SM5808.png]
